# Home-Based Virtual Reality Exercise and Resistance Training for Enhanced Cardiorespiratory Fitness in Community-Dwelling Older People with Sarcopenia: A Randomized, Double-Blind Controlled Trial

**DOI:** 10.3390/life15070986

**Published:** 2025-06-20

**Authors:** Chanakan Chitjamnogchai, Kornanong Yuenyongchaiwat, Natsinee Sermsinsaithong, Wararat Tavonudomgit, Lucksanaporn Mahawong, Sasipa Buranapuntalug, Chusak Thanawattano

**Affiliations:** 1 Department of Physical Therapy, Faculty of Allied Health Sciences, Thammasat University, 99 Moo 18, Paholyothin Road, Klong Luang, Rangsit 12120, Thailand; 2 Thammasat University Research Unit, Physical Therapy in Respiratory and Cardiovascular Systems, Thammasat University, Pathum Thani 12120, Thailand; 3 Biomedical Electronics and Systems Research Team, Assistive Technology and Medical Devices Research Group, National Electronics and Computer Technology Center, Pathum Thani 12120, Thailand

**Keywords:** sarcopenia, older people, cardiorespiratory, exercise

## Abstract

**Background**: Sarcopenia is characterized by low muscle mass and strength, as well as impaired physical performance. Older adults with sarcopenia experience decreased cardiorespiratory fitness. Physical exercise is recommended for the prevention and treatment of sarcopenia. Virtual reality (VR) exercise was introduced to improve physical activity. However, the effect of VR on cardiorespiratory function in older adults with sarcopenia has not been fully explored. This study aimed to explore the effects of home-based VR aerobic exercise combined with resistive exercise on cardiorespiratory performance in community-dwelling older adults with sarcopenia. **Subjects and Methods**: In a randomized controlled trial, 53 older adults with sarcopenia were divided into a home-based VR (n = 26) and a control group (CG; n = 27). The VR program combined aerobic and resistance exercises, performed three times per week for 12 weeks, while the CG received knowledge regarding the benefit of exercise and continued with their regular daily activities. All participants were required to undergo respiratory muscle strength and functional capacity tests before and after the 12-week intervention. Two-way mixed repeated ANOVA was conducted to compare within and between groups in cardiorespiratory performance. **Results**: The home-based VR exercise group showed significant improvement in pre-post (i.e., maximal inspiratory pressure (12.96 ± 1.49 cmH_2_O), maximal expiratory pressure (13.73 ± 1.72 cmH_2_O), functional capacity (28.32 ± 3.48 m), and between-group (maximal expiratory pressure (F (1,51) = 10.446, *p* = 0.002, np^2^ = 0.170). In contrast, the CG displayed a reduction in maximal expiratory pressure (−3.93 ± 1.69 cmH_2_O, *p* = 0.024) and functional capacity (−10.39 ± 3.42 m, *p* = 0.004) after the 12-week program. **Conclusions**: The home-based VR program provides older adults with sarcopenia an alternative exercise modality that can improve their cardiovascular performance.

## 1. Introduction

According to the World Health Organization, the number of people aged 60 years will increase to 1 in 6 people worldwide by 2030, and the number of older adults is anticipated to rise beyond 2.1 billion [1]. Sarcopenia is an age-related geriatric syndrome characterized by decreased skeletal muscle mass, muscle strength, and/or physical performance caused by aging or disease. Patients in their 70s have approximately 30–35% less skeletal muscle volume and muscle power, compared with those in their 40s [2]. Furthermore, muscle mass declines by 1–2% per year after the age of 50 [3].

The prevalence of sarcopenia among older community-dwelling Thai adults was 19.70% in 2019 and 21.46% in 2021, indicating that the 2-year incidence of sarcopenia was 2.44% [4]. Physical inactivity and biomarkers of functional performance are associated with sarcopenia in older adults [5,6]. Furthermore, elderly adults diagnosed with sarcopenia exhibit reduced respiratory muscle strength and cardiovascular endurance compared with non-sarcopenic older adults [7]. A systematic review and meta-analysis of randomized controlled trials demonstrated that exercise as a non-pharmacological intervention produced significant physiological and health benefits and prevented and/or delayed the onset of sarcopenia [8]. Moreover, high-quality evidence has demonstrated that the combination of resistance exercise and aerobic training leads to intermediate effectiveness as a therapy for enhancing physical performance compared with the individual effects of resistance or aerobic training alone [9].

Although traditional interventions such as aerobic and resistance exercise have shown effectiveness in enhancing physical performance and cardiovascular function in older individuals [10,11,12], older people face a barrier to exercise [13]. Factors associated with barriers to physical exercise in older people are often the following: physical disabilities, health conditions, lack of motivation, and time [14]. Consequently, there is growing interest in innovative, engaging, and accessible methods to support exercise in the aging population.

The use of technology to enhance adherence to physical exercise has been reported in several studies, with evidence suggesting that technology-based exercise programs may be more positive in promoting adherence compared to traditional exercise programs [15].

Virtual reality (VR) exercise is an innovative technology that immerses users in a computer-generated, multisensory, three-dimensional world in which they interact with a virtual environment using headgear and/or exercise devices. Additionally, VR-based exercise is more enjoyable and engaging than traditional exercise. In addition, the use of real-time feedback in virtual reality exercise promotes improvements in both cognitive function and physical abilities in older people [16]. Therefore, participants may have been more intrinsically motivated to engage in it.

VR-based exercise can enhance engagement in physical activity and reduce sedentary habits [17]. Although VR technologies are increasingly being explored in geriatric rehabilitation in terms of physical performance and muscle strength [18,19], the specific impact of VR interventions on cardiorespiratory performance in older adults with sarcopenia remains poorly understood. Therefore, this study aimed to explore the effects of VR exercise on the cardiopulmonary performance of community-dwelling older adults with sarcopenia.

## 2. Subjects and Methods

### 2.1. Participants

Older male and female adults aged 60–80 years were diagnosed with sarcopenia according to the Asian Working Group for Sarcopenia (AGWS) criteria [20]. Sarcopenia was defined as low muscle mass, low muscle strength, and/or slow gait speed. The appendicular skeletal muscle mass index was assessed using bioelectrical impedance analysis (BIA: Omron Body Composition Monitor HBF-375, Kyoto, Japan); a score of <7.0 kg/m^2^ for males or <5.7 kg/m^2^ for females was categorized as low muscle mass. Hand grip strength was assessed using a hand grip dynamometer (Takei Grip-D T.K.K.5101, Tokyo, Japan); cutoff scores < 28 kg for males or <18 kg for females were defined as low muscle strength. Finally, gait speed was measured using a 6 m walk test, and a gait speed < 1 m/s was defined as slow gait speed.

Using the G-power 3.1.9.4 program, the sample size was determined as 40 based on an effect size of 0.2308 [18], a statistical power of 0.80, and a significance level of 0.05. To account for potential data loss or technical errors, a minimum of 52 community-dwelling older adults with sarcopenia were enrolled in the study. Participants with no history of musculoskeletal problems, such as painful knee osteoarthritis and lower limb bone deformity, and neurological conditions (e.g., stroke) were included. Individuals who had cognitive impairment (assessed by the Montreal Cognitive Assessment (MoCA)-Thai version [21], score < 24 points) were excluded. The dependent variables are the respiratory muscle strength and functional capacity, while the independent variables are virtual home-based aerobic exercise combined with resistance training. In addition, the confounding variables are cognitive performance, body mass index, and physical activity.

All the enrolled participants were clustered and randomized into two groups (home-based VR and control). The study adopted a double-blind controlled trial design and a simple randomized sampling technique using the lottery method. The patients were trained using the VR exercise program.

### 2.2. Intervention Program

Home-based VR exercises relied on two main components: VR hardware and exercise software.

The VR hardware consisted of the following:

- MiniPC Android board, a board used to install the software
- Wireless Dance Mats Pad (OSTENT USB Non-Slip Dancing Step Dance Mat Pad Blanket, Mainland China; size: 930 × 830 × 80 mm)
- Revolution Non-Slip Footprint Mat connected to a PC through USB
- Air Mouse G10S (wireless 2.4 G): remote control
- Screen with HDMI capability (TV/Computer/Notebook screen size > 15 in) to show the VR coach and monitor heart rate on the screen.
- Polar Verity Sense OH1 (Optical Heart Rate Sensor: Polar Electro, Kempele, Finland)
- ANT+™: Armband (Cochrane, AB, Canada), connected through Bluetooth to monitor heart rate in real-time while exercising within a suitable range with the user-specific exercise program.

**Figure 1:**
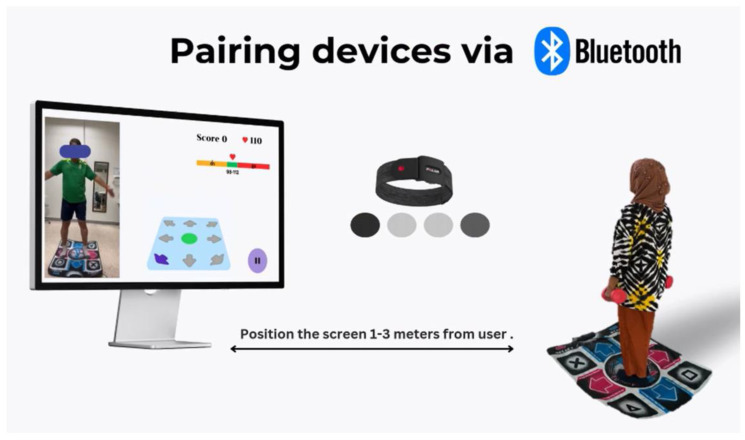
A component of virtual reality exercise.

Setting: The screen was set at a distance of approximately 1–3 m from the participant and maintained at a distance of approximately 3–5 m between the devices (Figure 1).

The software was an Android system that served to connect Polar OH1 devices with Bluetooth to display and monitor heart rate while exercising. For the aerobic training, heart rate from Polar–OH1 was shown on the screen during exercise and adjusted by increasing or decreasing the speed to achieve the target heart rate (moderate intensity exercise: 40–59% heart rate reserve). The exercise program started with a 5-min leading video to serve as a warm-up, while the user followed along with the video and continued with 30 min of aerobic training at a target heart rate of 40–59% of heart rate reserve. Following aerobic training, there was a 20-min resistance training in which the participants used dumbbell weights (60–70% 1RM). Finally, the program concluded with a 5-min cool-down period. The participants performed home-based VR exercises for a total of 60 min. The VR exercise protocol was modified from previous studies [22,23,24] and was approved by physical therapists with >10 years of experience (Supplement Table S1). The screen (screen size > 15 inches) was set at a distance of approximately 1–3 m from the participants. A combination of resistance and aerobic training was performed three times per week for 12 weeks. Exercise intensity was set at 40–59% of heart rate reserve and a rate of perceived exertion (RPE) on a Borg scale of less than 13 out of 20. The control group (CG) received knowledge about the benefits of exercise and was requested to perform their usual activities throughout the study.

All participants were required to undergo respiratory muscle strength and cardiovascular endurance tests before and after the 12-week intervention. Respiratory muscle strength measures included maximal inspiratory pressure (MIP) and maximal expiratory pressure (MEP). Participants were instructed to exhale fully to the residual volume and then breathe in deeply and hold for 1.5 s; this was defined as MIP. MEP was defined as inhaling deeply until the total lung capacity was reached and then exhaling quickly and holding for 1.5 s. All participants repeated the MIP and MEP measurements three to five times, and the maximum values were recorded. Details on respiratory muscle strength were obtained according to the recommendations of the American Thoracic Society/European Respiratory Society International [25].

The 6 min walk test (6MWT), which is a submaximal exercise test, was used as a measure of cardiovascular endurance [26]. The test was conducted according to a standardized recommendation from the American Thoracic Society statement [26]. The participants were required to walk for 6 min in a 30-m corridor and the total walking distance was recorded. After 12 weeks, the participants were invited to assess their cardiorespiratory performance.

### 2.3. Statistical Analysis

The demographic and clinical characteristics of the individuals were presented as descriptive statistics. The Shapiro–Wilk test was conducted to analyze the normality of data distribution. A two-way mixed repeated ANOVA (group [2] × time [2]) with a Bonferroni post hoc test was performed to analyze the effects of home-based VR exercise on the 6MWT and respiratory muscle strength. The IBM^®^ SPSS^®^ Statistics version 27 was used, and statistical significance was set at *p*-value < 0.05.

## 3. Results

**Figure 2:**
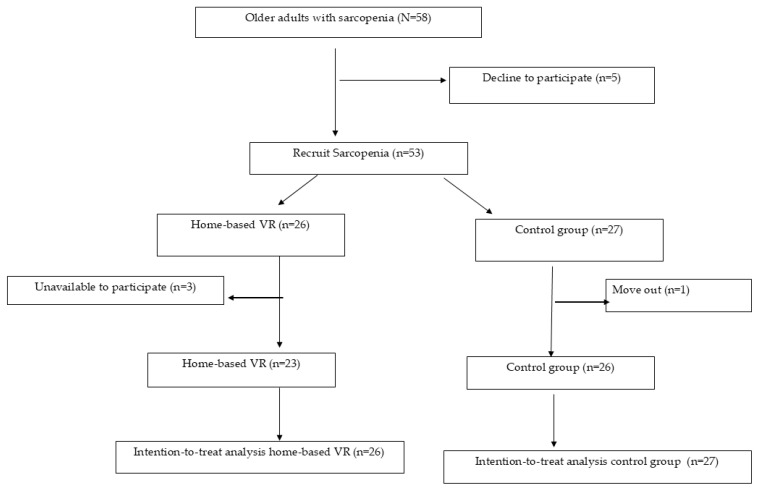
Flow chart of the recruitment procedure and the study profile of participants.

Fifty-eight individuals were defined as having sarcopenia according to the Asian Working Group of Sarcopenia (AWGS) 2019 version. Ten participants refused to participate; hence, a total of 53 individuals were enrolled in this study. Forty-nine participants (VR, n = 23, CG, n = 26) completed the 12-week intervention period. Four participants were unavailable to complete the study: 1 from the CG, who was reallocated, and 3 from the VR group (1 was unavailable, and 2 had knee pain during the intervention program). There were no significant differences in baseline age, sex, cardiorespiratory performance between those who completed the intervention and those who did not (*p* > 0.05; see Table 1). Therefore, an intention-to-treat analysis was performed (Figure 2).

Table 1 compares the participant characteristics between the VR and the CG. There were no statistically significant differences in participant characteristics between the VR and the CG regarding sex, comorbidities, age, and cardiorespiratory performance at baseline (*p* > 0.005).

Table 2 shows a comparison of the effects of VR on cardiorespiratory performance. There were statistically significant between-group differences in MEP F (1,51) = 10.446, *p* = 0.002, np^2^ = 0.170) changes from the pre- to the post-12-week period, in which the VR group had higher MIP values than the CG. Compared with the baseline period, improved cardiorespiratory performance (i.e., respiratory muscle strength and functional capacity) was noted in the VR group (*p* < 0.05). While statistically significant improvements in MEP (Δ13.73 cmH_2_O, *p* < 0.001) and 6MWT (Δ28.32 m, *p* < 0.001) were observed in the VR group after the 12-week intervention, the CG showed a significant decrease (*p* < 0.05). Furthermore, statistically significant improvements in MIP (Δ12.96 cmH_2_O, *p* < 0.001) were observed in the VR group but not in the CG (*p* > 0.05).

## 4. Discussion

Increasing respiratory muscle strength and 6-MWD was observed in home-based VR over a 12-week intervention, indicating the effects of home-based VR-enhanced cardiorespiratory performance in older adults with sarcopenia. Respiratory function naturally declines owing to physiological changes associated with aging, such as diaphragm thinning and the loss of respiratory muscle strength [27]. In older adults with sarcopenia, there is typically a thinner diaphragm and lower values for MIP and MEP, mainly due to a decline in diaphragm muscle strength [7,28]. Overall, sarcopenia appears to affect the diaphragm muscle by causing muscle fiber atrophy and weakness, with fiber type-specific effects that particularly impair the high force-generating fibers (i.e., type IIx and/or IIb fibers) [29]. However, the present study did not assess the thickness of the diaphragm; therefore, further studies should be explored.

MIP serves as a key indicator of inspiratory muscle strength. Previous studies have demonstrated that exercise with resistance improved respiratory muscle strength in older adults [30,31]. Further, a comparison between peripheral resistive training and respiratory muscle training showed that resistance training improves MIP and MEP in older people with sarcopenia [32]. This finding indicates that physical exercise or activity with resistive training could improve respiratory muscle strength.

According to the American Thoracic Society and the European Respiratory Society guidelines on respiratory muscle testing, an MIP of 80 cmH_2_O is considered sufficient to rule out clinically significant inspiratory muscle weakness [25]. Although the VR group showed an increase in MIP after 12 weeks of exercise, the mean values remained below 80 cmH_2_O, indicating persistent inspiratory muscle weakness.

Regarding the expiratory muscle, MEP reflects the strength of the abdominal and other expiratory muscles [33]. The VR group showed an increase in MEP after 12 weeks of exercise. The improvement suggests enhanced expiratory muscle strength following the intervention. The study proposes that intense physical exercise activities activate the abdominal muscles, raising abdominal pressure. This pressure leads to tension in the diaphragm, which reduces the transfer of intra-abdominal pressure to the thorax, thereby strengthening the respiratory muscles [34]. Strengthening of the abdominal and intercostal muscles improves MEP by enhancing expiratory force and airway clearance [34]. Lee et al. suggest that squat exercises effectively develop lower limb muscle strength by activating the quadriceps, hamstrings, gastrocnemius, and gluteus maximus [35]. In this study, the resistance training program included squat exercises, which may have contributed to increased abdominal muscle strength, potentially leading to improvements in MEP. Additionally, moderate-intensity aerobic exercise has been shown to enhance lung capacity, endurance, and overall respiratory function [35].

This study involved both resistance and aerobic training, where aerobic training promotes an increase in Type I muscle fibers, enhancing fatigue resistance and oxidative capacity, while resistance training promotes an increase in Type IIa muscle fibers, improving power and speed capabilities [36]. These factors contribute to the enhanced strength of respiratory muscles in older adults with sarcopenia following exercise. In the present study, respiratory muscle strength increased after exercise with home-based VR training; however, these remained within the range of respiratory weakness. The benefits of respiratory muscle training have been reported in older adults, including a strengthened diaphragm, improved aerobic capacity, enhanced respiratory muscle strength, and better coughing ability [37]. Therefore, future studies should consider incorporating respiratory muscle training as part of the exercise program for enhanced respiratory function.

Home-based aerobic exercise with moderate-intensity exercise plus resistive training increased cardiovascular endurance. Previous studies reported that 6MWT was significantly reduced in individuals with sarcopenia [7,38]. Additionally, the minimum clinically important difference (MCID) for the 6MWT distance in older adults is 17.8 m [39]. In this study, the average change in walking distance was approximately 32.02 m, which meets the threshold for clinically significant improvement. Therefore, engaging in home-based VR aerobic exercise and resistance training significantly enhances cardiovascular endurance in older adults with sarcopenia, with both clinically meaningful and statistically significant effects.

In the CG, a slight decline in cardiorespiratory performance was observed over 12 weeks; MIP dropped by 0.33 cmH_2_O, MEP decreased by 3.93 cmH_2_O, and 6MWT reduced by 10.39 m. This study revealed a reduced cardiorespiratory performance in older people with sarcopenia. Therefore, physical exercise has been demonstrated to have positive effects on managing sarcopenia and helping to prevent increased comorbidity in older adults [40]. Additionally, aerobic training induces peak oxygen consumption [41], and physical exercise has been linked to reduced risk of sarcopenia in older people [42]. Mitochondrial dysfunction is one of the mechanisms of age-related sarcopenia; hence, beneficial aerobic exercise, resistance, or combination exercise training can improve ATP production in mitochondria and ultimately enhance cardiovascular endurance [43]. 

### Strengths and Limitations

The strength of the current study lies in its investigation of the effect of home-based VR aerobic exercise and resistance training on respiratory muscle strength and cardiovascular endurance in community-dwelling older adults with sarcopenia. The study design was a double-blind, randomized controlled trial. Additionally, we developed the innovative home-based VR exercise, and the exercise protocol was adapted from evidence-based support and reviewed by expert physical therapists. The study had some limitations; older adults with sarcopenia were required to be able to walk independently. Consequently, these results might not be generalizable to the general population, including those with severe mobility impairments. Additionally, the CG received only educational information and continued their regular daily activities, which might limit comparison or potential bias due to an inactive CG. Therefore, active control conditions might provide a better comparison. The intervention was conducted over a 12-week period; however, the long-term sustainability of the effects of home-based VR aerobic exercise combined with resistance training was not assessed. Future studies should include follow-up assessments to determine whether the benefits of the intervention are maintained over time. Finally, the study did not measure compliance or adherence to the home-based program of VR intervention, which represents a limitation. Therefore, the findings of the study should be interpreted with caution; it may be the case that affects the practical implementation of a home-based VR program, in particular in older adults with sarcopenia.

## 5. Conclusions

This study reveals that VR exercise and resistance training, performed 3 days per week for 3 months, improve cardiorespiratory performance in older adults with sarcopenia, compared with the CG. Therefore, regular exercise is crucial for managing sarcopenia and improving cardiorespiratory performance. In contrast, inactivity is linked to decreased cardiorespiratory performance. Therefore, home-based VR aerobic exercise with resistance training can serve as a valuable approach for improving respiratory muscle strength and cardiovascular endurance in older people with sarcopenia.

## Figures and Tables

**Table 1:** General Characteristics data, cardio-respiratory performance in the home-based VR and the control group.

	Virtual Reality Group (n = 26)	Control Group (n = 27)	x^2^	*p*-Value
Sex			0.092 (1)	0.761
Male (%)	4 (44.44)	5 (55.56)		
Female (%)	22 (50.00)	22 (50.00)		
Diabetic mellitus (%)	12 (66.67)	6 (33.33)	3.382	0.066
Hypertension (%)	15 (60.00)	10 (40.00)	2.268	0.132
Dyslipidemia (%)	3 (50.00)	3 (50.00)	0.002	0.961
Heart disease (%)	1 (20.00)	4 (80.00)	1.865	0.172
	**Mean ± SD**	**Mean ± SD**	***t* (test)**	***p*-Value**
Age (years)	69.23 ± 4.79	71.15 ± 5.94	−1.290	0.203
Maximal inspiratory pressure (cmH_2_O)	45.27 ± 21.06	51.93 ± 24.09	−1.069	0.290
Maximal expiratory pressure (cmH_2_O)	44.31 ± 15.93	47.48 ± 14.58	−0.757	0.453
6 min walk test (meters)	334.52 ± 46.96	354.48 ± 70.37	−1.210	0.232

**Table 2:** Comparison of the effects of virtual home-based aerobic exercise on cardio-respiratory performance and depression in older people with sarcopenia after a 12-week intervention program.

	Virtual Reality Group (n = 26)		Change Mean ± SE VR vs. Control	Significant Within VR gr	Control Group (n = 27)		Change Mean ± SE	Significant Within CG	*p*-Value VR vs. Control	Significant Between VR gr and CG
	Pre-Test	Post-Test			Pre-Test	Post-Test				
MIP (cmH_2_O)	45.27 ± 21.06	58.23 ± 20.21	12.96 ± 1.49 ***	F(1,51) = 75.593, *p* < 0.001, np^2^ = 0.597	51.93 ± 24.09	51.59 ± 23.19	−0.33 ± 1.46	F(1,51) = 0.052, *p* = 0.821, np^2^ = 0.001	0.272	F(1,51) = 1.231, *p* = 0.272, np^2^ = 0.024
MEP (cmH_2_O)	44.31 ± 15.93	58.04 ± 19.81	13.73 ± 1.72 ***	F(1,51) = 63.759, *p* < 0.001, np^2^ = 0.556	47.48 ± 14.58	43.56 ± 12.01	−3.93 ± 1.69 *	F(1,51) = 5.413, *p* = 0.024, np^2^ = 0.096	0.002	F(1,51) = 10.446, *p* = 0.002, np^2^ = 0.170
6MWT (meter)	334.52 ± 46.96	362.84 ± 39.46	28.32 ± 3.48 ***	F(1,51) = 66.104, *p* < 0.001, np^2^ = 0.564	354.48 ± 70.37	344.09 ± 39.46	−10.39 ± 3.42 **	F(1,51) = 9.238, *p* = 0.004, np^2^ = 0.153	0.229	F(1,51) = 1.479, *p* = 0.229, np^2^ = 0.028

* *p* < 0.05, ** *p* < 0.01, *** *p* < 0.001, VR: virtual reality, gr: group, CG: control group, SE: standard error, MIP: Maximal inspiratory pressure, MEP: Maximal expiratory pressure, 6MWT: 6 min walk test.

## Data Availability

The original contributions presented in this study are included in the article. Further inquiries can be directed to the corresponding author.

## Supplementary Materials

The following supporting information can be downloaded at: https://www.mdpi.com/article/10.3390/life15070986/s1, Table S1: Home-based virtual reality exercise program.
