# Supplementary material for: Home-Based Virtual Reality Exercise and Resistance Training for Enhanced Cardiorespiratory Fitness in Community-Dwelling Older People with Sarcopenia: A Randomized, Double-Blind Controlled Trial
Source: Life (Basel). 2025 Jun 20;15(7):986. doi: 10.3390/life15070986 (PMC12299642; doi:10.3390/life15070986)
Supplement: Supplementary file 1 [file life-15-00986-s001.zip › life-3628762-supplementary.pdf]

Supplement Table 1: Home-based virtual reality exercise program

VR exercise program for week 1-6		VR exercise program for week 7-12	
**Warm up (5 minutes)**<br>Trunk lateral flexion, knee extension, shoulder abduction, high knee, marching	10 reps/ set, 3 set	**Warm up (5 minutes)**<br>Trunk lateral flexion, knee extension, shoulder abduction, high knee, marching	10 reps/ set, 3 set
**Aerobic training** (5 exercise, 5 cycle = 30 minutes)<br>1.Hip abduction-adduction with shoulder abduction-adduction<br>2.Hip flexion-extension<br>3.Mini squat-knee flexion<br>4.Hip flexion with knee extension<br>5.Boxing	Frequency: 3 days/week, Intensity: 40-59% HRR	**Aerobic training** (3 exercise, 3 cycle = 31 minutes)<br>1.Hip abduction-adduction with shoulder abduction-adduction<br>2.Hip flexion-extension<br>3.Mini squat-knee flexion<br>4.Hip flexion with knee extension<br>5.Boxing<br>6.Uppercut<br>7.Hip flexion-adduction<br>8.shoulder abduction with hip abduction<br>9.push forward with mini lunge	Frequency: 3 days/week, Intensity: 40-59% HRR
**Resistance training with dumbbell**<br>1.Mini squat with knee flexion<br>2.Mini forward lunge<br>3.Calf raise<br>4.Side lunge<br>5.Biceps curl<br>6.D2 flex<br>7.Shoulder press	10 reps/set, 3 sets	**Resistance training with dumbbell**<br>1.Mini squat with knee flexion<br>2.Mini forward lunge<br>3.Calf raise<br>4.Side lunge<br>5.Biceps curl<br>6.D2 flex<br>7.Shoulder press	10 reps/set, 3 sets
**Cool down (5 minutes)**<br>Marching<br>Stretching exercise	10 reps/set, 3 sets	**Cool down (5 minutes)**<br>Marching<br>Stretching exercise	10 reps/set, 3 sets
